# Supplementary material for: Genome-wide association study on color-image-based convolutional neural networks
Source: PeerJ. 2025 Jan 13;13:e18822. doi: 10.7717/peerj.18822 (PMC11737327; doi:10.7717/peerj.18822)
Supplement: Supplemental Information 1 [file peerj-13-18822-s001.docx]

**S1 Table. Group P001 matched genes.**

| **SNP** | **Gene** | **SNP** | **Gene** | **SNP** | **Gene** |
| --- | --- | --- | --- | --- | --- |
| rs11248915 | FAM234A | rs2431322 | PAM | rs2744374 | DSP |
| rs10873395 | TTC5 | rs930465 | ASTN2 | rs1402656 | GLP2R |
| rs2108978 | AKAP10 | rs2878176 | METTL24 | rs10893668 | LINC02712 |
| rs1358169 | THSD7A | rs1978137 | THSD7A | rs2287144 | TOX3 |
| rs765534 | LINC02756 | rs3943074 | SUMF1 | rs10809439 | LOC105375974 |
| rs17710561 | MYRIP | rs2439614 | KLHL1 | rs2861579 | TLL2 |
| rs2989476 | LINC01748 | rs4689713 | SORCS2 | rs8299 | ELP2 |
| rs4845690 | LOC105371449 | rs1874237 | LOC101928978 | rs835070 | LOC107986376 |
| rs4820258 | NCF4 | rs2175334 | LOC105373417 | rs6755520 | LOC105373742 |
| rs11686149 | PCGEM1 | rs7785392 | LAMTOR4 | rs10769118 | TRIM5 |
| rs11166823 | FAM135B | rs262998 | PARL | rs7016691 | NRG1 |
| rs7804595 | LOC105375170 | rs302946 | UBE2K | rs951366 | NUCKS1 |
| rs2381971 | PTPRD | rs9692306 | LOC105375523 | rs6795481 | SUMF1 |
| rs4519393 | MC2R | rs4521284 | ROBO2 | rs41496145 | LRGUK |
| rs17109102 | NRXN3 | rs1537136 | ADAMTSL1 | rs238328 | DGKH |
| rs2786864 | MAN1C1 | rs16971269 | DNAH17 | rs7967385 | LINC02463 |
| rs10185950 | RND3 | rs6589847 | GRIK4 | rs9805321 | TSC22D1 |
| rs4643515 | TNS1 | rs7039172 | SLC24A2 | rs17383719 | PBX1 |
| rs26999 | MCTP1 | rs521069 | MPPED2 | rs8013657 | FOXN3 |
| rs7575017 | HECW2 | rs7781054 | RPA3 | rs971215 | SNAP91 |
| rs9945680 | ZBTB14 | rs325923 | CEPT1 | rs1520332 | RGS6 |
| rs1111571 | PRMT7 | rs39700 | MAST4 | rs1519254 | MYOCD |
| rs4981951 | TTC5 | rs2935651 | CTBP2 | rs2608631 | GSTA2 |
| rs12325711 | SHISA6 | rs2278082 | CCDC93 | rs9326151 | CYP4X1 |
| rs25112 | SYN3 | rs1468291 | SERGEF | rs6800127 | SOX2-OT |
| rs10503913 | NRG1 | rs2965819 | ANKRD11 | rs4460877 | TMEM132D |
| rs1946127 | RBFOX1 | rs41462346 | LOC105377488 | rs835087 | LOC107986376 |
| rs10908752 | SLAMF9 | rs16932962 | TTC39B | rs2419855 | NRAP |
| rs10780661 | SLC28A3 | rs10846883 | TMEM132B | rs1949560 | RGS6 |
| rs4783558 | SMPD3 | rs41322345 | LYRM1 | rs2741919 | MEFV |
| rs1579808 | RIC8B | rs908916 | LOC100287010 | rs9848209 | DCUN1D1 |
| rs1817116 | LOC102724419 | rs11687426 | DNAH6 | rs11971451 | LRGUK |
| rs8067774 | RHBDF2 | rs6935425 | LOC105374898 | rs743904 | TNR |
| rs11104913 | KITLG | rs4256218 | SCD5 | rs3121465 | NRAP |
| rs6019582 | ARFGEF2 | rs4797825 | MC2R | rs11888862 | LINC01102 |
| rs757747 | SEMA3A | rs11237796 | TENM4 | rs10490051 | SLC8A1 |
| rs12108894 | LOC105378967 | rs4980083 | ZCCHC24 | rs10758270 | FAM205A |
| rs12213597 | FAM184A | rs12125340 | ANKRD35 | rs4438608 | FGF12 |
| rs12070036 | ZNF678 | rs2453552 | MOB3B | rs11101442 | WDFY4 |
| rs4077060 | PLXDC1 | rs2323587 | BARX2 | rs4640066 | LOC105370265 |
| rs687706 | PPARGC1A | rs2744778 | MAN1C1 | rs8082727 | LOC107985179 |
| rs6443099 | GRM7 | rs2429423 | TANC2 | rs10821716 | ANK3 |
| rs17817736 | ABCC5 | rs2097657 | MYH3 | rs10246251 | OR6B1 |
| rs4394984 | LOC728755 | rs10780196 | CACNA1B | rs4916899 | TMEM161B-AS1 |
| rs361021 | IQSEC1 | rs3806028 | COL19A1 | rs12889349 | AKAP6 |
| rs3864206 | SORCS2 | rs9322991 | LINC00639 | rs6676208 | PACC1 |
| rs2744785 | MAN1C1 | rs10494520 | AXDND1 | rs2013111 | LOC105377136 |
| rs16822807 | LINC02006 | rs2429427 | TANC2 | rs10914427 | LINC01226 |
| rs4149269 | ABCA1 | rs11855354 | IDH3A | rs901130 | CCDC33 |
| rs1555890 | TGFBR3 | rs16968438 | ASIC2 | rs4708575 | LOC105378145 |
| rs3956806 | KSR2 | rs7971798 | LOC101928387 | rs1968059 | THSD7A |
| rs1681464 | PDGFD | rs16897057 | SMILR | rs1490879 | LINC02438 |
| rs17128052 | GCH1 | rs4560424 | LOC102724210 | rs7838570 | LOC105375821 |
| rs11627566 | AKAP6 | rs2607872 | GDF10 | rs12367370 | LINC02373 |
| rs2521760 | GSDME | rs159349 | MCTP1 | rs333950 | CSF1 |
| rs703277 | LOC101928196 | rs5754259 | SYN3 | rs8052274 | LINC02126 |
| rs2082622 | PARD3B | rs4817721 | LOC100506403 | rs16968964 | AANAT |
| rs9822318 | SENP7 | rs12432214 | LINC00639 | rs1026330 | LDLRAD4 |
| rs1294623 | COL28A1 | rs13398112 | LOC105374786 | rs6056855 | PAK5 |
| rs11902447 | LINC01293 | rs2242663 | CTSF | rs4652840 | LOC105378641 |
| rs6974119 | COL28A1 | rs1894521 | PHF21B | rs2225889 | GPC6 |
| rs7153625 | NRXN3 | rs9557205 | UBAC2 | rs700469 | PKP1 |
| rs12539880 | CRYGN | rs445237 | LINCR-0002 | rs913785 | MACROD2 |
| rs1863967 | ADAMTS16 | rs17016168 | SNCA | rs309307 | RNF144A |
| rs17188506 | KCNH1 | rs1683348 | FHIT | rs6932855 | FAM229B |
| rs7582590 | LOC105369168 | rs659991 | DGKH | rs357897 | DYM |
| rs12732979 | ADGRL2 | rs16905104 | LOC102724670 | rs1410643 | DCLK1 |
| rs949981 | MIR3681HG | rs8032618 | IDH3A | rs4073987 | NRG3 |
| rs7925917 | C11orf53 | rs1748041 | PADI4 |  |  |

**S2 Table. Group P005 matched genes.**

| **SNP** | **Gene** | **SNP** | **Gene** | **SNP** | **Gene** |
| --- | --- | --- | --- | --- | --- |
| rs9430155 | SLC25A33 | rs544276 | LPAR1 | rs11214512 | NCAM1 |
| rs10771012 | SOX5 | rs12757229 | NEGR1 | rs2482814 | PCDH15 |
| rs984066 | DPP10 | rs17822638 | LOC105375751 | rs6587041 | MTMR7 |
| rs2240920 | ITIH3 | rs4480236 | AUH | rs5024299 | PIEZO2 |
| rs4765905 | CACNA1C | rs745696 | ZNF219 | rs17782224 | PLEKHH1 |
| rs3890011 | CYP4A11 | rs4984997 | ABAT | rs3790999 | COL6A3 |
| rs4973362 | SPATA3 | rs11259557 | FAM171A1 | rs620498 | JADE2 |
| rs1439651 | PCGEM1 | rs2092867 | NFIA | rs2492634 | LOC105373122 |
| rs11626813 | ZNF410 | rs2197714 | EPHB1 | rs7181486 | IREB2 |
| rs10490726 | LOC105374786 | rs514931 | ZNF407 | rs13149391 | SPATA5 |
| rs4568670 | PTBP3 | rs12433867 | RIN3 | rs12705305 | SRPK2 |
| rs4968762 | TANC2 | rs11178364 | PTPRR | rs329674 | IGSF9B |
| rs3815416 | PNLIPRP2 | rs1891395 | KCNQ5 | rs2267023 | BCR |
| rs2042794 | SYN3 | rs4263970 | EPHB2 | rs16966655 | SPRED1 |
| rs11250723 | ADARB2 | rs2694696 | ACTG2 | rs3816133 | TGFBRAP1 |
| rs1016351 | CWF19L2 | rs1861474 | MSI2 | rs1051055 | CDC123 |
| rs12703567 | OR2A12 | rs11720582 | ADAMTS9-AS2 | rs11612882 | RIMKLB |
| rs6856389 | OPRPN | rs10235703 | COBL | rs6728886 | ITGA4 |
| rs4560424 | LOC102724210 | rs1520884 | HPS5 | rs3858691 | C12orf42 |
| rs7899110 | RBM17 | rs17420654 | LOC105374505 | rs3862148 | EPN2 |
| rs1579808 | RIC8B | rs7544440 | GREM2 | rs321908 | ZNF766 |
| rs10929168 | ASB18 | rs4509530 | LOC105378798 | rs17100876 | RHOJ |
| rs1817116 | LOC102724419 | rs41346444 | GRM7 | rs11021901 | GALNT18 |
| rs5747087 | CECR2 | rs2494248 | LINC00853 | rs2756115 | CUTC |
| rs8007791 | CCDC88C | rs7475474 | LOC105378516 | rs2111476 | CCDC85A |
| rs8008996 | CCDC88C | rs11900907 | CERS6 | rs907183 | MFHAS1 |
| rs1469853 | FGF14 | rs5765490 | FBLN1 | rs6750599 | BCL2L11 |
| rs12706898 | NRF1 | rs9295618 | DCDC2 | rs41507647 | TMEM178B |
| rs41361356 | CDRT1 | rs9555336 | FAM155A | rs16984549 | GRIK1 |
| rs11903030 | LOC105374822 | rs11679769 | OTOF | rs12598711 | LOC102724859 |
| rs1574768 | ZDHHC21 | rs2834238 | DONSON | rs10506625 | TSPAN8 |
| rs16883315 | LOC107986178 | rs6434182 | ZC3H15 | rs1013511 | LOC102723803 |
| rs2594135 | FHIT | rs5747145 | CECR2 | rs16872235 | GFM2 |
| rs193858 | CDHR3 | rs543735 | CSMD2 | rs7780487 | DPP6 |
| rs11997 | LOC105370401 | rs6556858 | MCTP1 | rs16857239 | CACNA1E |
| rs10989064 | TEX10 | rs7972185 | MGAT4C | rs11090417 | MYO18B |
| rs10838807 | LOC112268071 | rs661438 | LOC105378143 | rs9533328 | EPSTI1 |
| rs7192050 | LOC107984894 | rs41500746 | LOC101927369 | rs13438514 | SKAP2 |
| rs11058480 | RSRC2 | rs17167543 | LRGUK | rs2031727 | ZFYVE28 |
| rs228787 | TMEM101 | rs9313968 | LOC105377700 | rs16897440 | VPS13B |
| rs2269195 | COX15 | rs41527748 | ARHGAP24 | rs10458681 | PLXDC2 |
| rs7130241 | OPCML | rs9483454 | STX7 | rs1368085 | LOC105373730 |
| rs9397747 | SCAF8 | rs6593171 | GRB10 | rs847317 | RGS6 |
| rs623823 | JAM3 | rs636391 | GAREM1 | rs526501 | ADGRB3 |
| rs2231963 | TRIM68 | rs12166809 | PACSIN2 | rs655970 | KMO |
| rs930758 | UCHL1 | rs2039342 | LINC00402 | rs10754821 | LOC105373262 |
| rs17503541 | MSH2 | rs4287138 | CACNA1E | rs2190998 | PDE1C |
| rs11893024 | FSIP2 | rs13416248 | ADD2 | rs760233 | TTLL5 |
| rs17155593 | FAM107B | rs7984662 | GPC5 | rs7638004 | CRYBG3 |
| rs10756202 | LOC102724027 | rs9900811 | CA10 | rs10037212 | CAST |
| rs13248141 | LOC107986920 | rs4401492 | TRAPPC11 | rs8176044 | KEL |
| rs1997034 | ETNK2 | rs9557193 | GPR183 | rs12233096 | HDAC4 |
| rs2292641 | PGS1 | rs9303394 | MSI2 | rs4656538 | POU2F1 |
| rs2867880 | CLEC16A | rs10806995 | RIPOR2 | rs16869564 | LOC107986432 |
| rs2744785 | MAN1C1 | rs11974205 | LINC01006 | rs1956143 | LOC105370461 |
| rs10779279 | ESRRG | rs12910386 | ACSBG1 | rs3809903 | LDLRAD4 |
| rs10002459 | TBC1D9 | rs2542294 | TSHZ3-AS1 | rs208021 | PCM1 |
| rs7146075 | NRXN3 | rs324139 | POLR1C | rs457587 | C5orf67 |
| rs11255267 | ITIH5 | rs12543538 | LINC01111 | rs10204 | PAGR1 |
| rs4765902 | CACNA1C | rs10218575 | LINC01725 | rs2574976 | SGMS1 |
| rs1400730 | ABLIM2 | rs7821050 | LRRC69 | rs4978934 | SVEP1 |
| rs3762503 | PRPF40A | rs7571692 | SPAG16 | rs4484586 | DNAH11 |
| rs621698 | JAM3 | rs11265432 | LOC105371468 | rs17753220 | TPM1 |
| rs466639 | RXRG | rs7971179 | FAR2 | rs1485854 | LOC105373628 |
| rs7782228 | LOC105375170 | rs12444562 | JPH3 | rs8030277 | LOC105370982 |
| rs6588759 | CHST15 | rs4672393 | BCL11A | rs1059502 | CPB1 |
| rs9472138 | POLR1C | rs11071450 | FAM81A | rs6977749 | PTN |
| rs4894769 | PLD1 | rs2471857 | DRD2 | rs7927234 | LGR4 |
| rs4519686 | SFMBT1 | rs7289981 | TPTEP2-CSNK1E | rs768354 | ITGA9 |
| rs514636 | LAMP3 | rs9908356 | CDRT8 | rs2220812 | LOC105370481 |
| rs11889699 | SLC39A10 | rs11178378 | PTPRR | rs2281719 | GALNT2 |
| rs2238056 | CACNA1C | rs2000526 | TBCEL | rs17614930 | LOC105371738 |
| rs1844908 | ZNF215 | rs12582879 | GRIP1 | rs76674 | BCL11A |
| rs10878331 | RPSAP52 | rs7229380 | LOC105371956 | rs134661 | KREMEN1 |
| rs1565073 | DOCK10 | rs6446159 | FHIT | rs10509362 | LRMDA |
| rs615837 | NDST3 | rs2836532 | ERG | rs7777879 | RPA3 |
| rs1179494 | FEZ2 | rs11140664 | LOC102724036 | rs1804690 | HYOU1 |
| rs269324 | SHISAL2A | rs494958 | SRD5A1 | rs4936749 | UBASH3B |
| rs11046115 | GYS2 | rs9850375 | ADCY5 | rs2371358 | LOC105377123 |
| rs17167639 | LRGUK | rs4112527 | ITGBL1 | rs41447349 | LOC105379315 |
| rs8050643 | CDH13 | rs6135208 | MACROD2 | rs8095171 | LOC105372185 |
| rs11687654 | LOC105369165 | rs615967 | RAD18 | rs7846088 | CPQ |
| rs2206509 | PAPPA2 | rs3737606 | PTPRU | rs930965 | RGS22 |
| rs7190365 | DYNLRB2-AS1 | rs17829626 | SCN3A | rs4308217 | CD86 |
| rs2812344 | FAM205A | rs1418953 | AGBL4 | rs1227053 | CDH23 |
| rs2466296 | SLC30A8 | rs12874185 | GPC5 | rs2834213 | IFNGR2 |
| rs12157657 | GUSBP11 | rs6425658 | STX6 | rs6774426 | LMCD1-AS1 |
| rs910050 | TSBP1 | rs955271 | LOC105378029 | rs4968775 | CYB561 |
| rs4632219 | LOC107985176 | rs4789291 | UBE2O | rs1549759 | CDK5 |
| rs9850123 | FGF12 | rs10954689 | PCLO | rs454182 | LOC107986586 |
| rs11676763 | TOGARAM2 | rs17257945 | SEC24D | rs6446716 | LOC101928306 |
| rs11015260 | PDSS1 | rs403029 | GATA3 | rs12363571 | PAX6-AS1 |
| rs6715049 | LOC102724542 | rs17315775 | SKAP2 | rs9679095 | COL4A3 |
| rs17083377 | FAM172A | rs2177191 | LOC105375343 | rs4974196 | ERC2 |
| rs4328757 | TRANK1 | rs7528364 | KIF26B | rs1667295 | B4GALT6 |
| rs10982716 | DELEC1 | rs6547544 | LOC107985905 | rs2460456 | SPG7 |
| rs742473 | PAPPA2 | rs2154487 | GRIK1 | rs2161139 | LOC107986381 |
| rs2289496 | TSPYL5 | rs1316326 | MAN1A2 | rs1872083 | SDK2 |
| rs10189164 | DTNB | rs2243834 | RAB10 | rs11935252 | TLR2 |
| rs17257773 | STXBP6 | rs17345702 | LOC105373531 | rs754960 | LINC00871 |
| rs2139594 | RGS6 | rs2488556 | VAV2 | rs11077154 | RBFOX1 |
| rs1030668 | TENM2 | rs1457947 | LOC105377862 | rs2793772 | LINC01039 |
| rs9482771 | RSPO3 | rs4742201 | UHRF2 | rs4580760 | SV2C |
| rs4293434 | MYO1D | rs10454502 | LOC105370002 | rs4413512 | ARHGEF28 |
| rs544158 | MAST4 | rs6976196 | KIAA1549 | rs586684 | EBF3 |
| rs702465 | BOLA3 | rs12928353 | MIR193BHG | rs3814821 | IFI27 |
| rs2945401 | KSR1 | rs2618129 | LOC102724710 | rs2884208 | HPCAL1 |
| rs703277 | LOC101928196 | rs2122576 | SMC2 | rs7997649 | LINC00572 |
| rs16957091 | CDAN1 | rs7117858 | LINC02751 | rs7833665 | LOC105375630 |
| rs12343877 | TMEM252-DT | rs580018 | LOC105376214 | rs6795436 | ITGA9 |
| rs4900764 | MDGA2 | rs17479963 | TMEM212 | rs11251392 | LOC105376350 |
| rs4705247 | LOC102546294 | rs8088134 | DLGAP1 | rs212857 | SKAP2 |
| rs943392 | FAM102A | rs6932648 | GLO1 | rs5761272 | MYO18B |
| rs36143 | MAST4 | rs2238202 | RGS6 | rs7972260 | TMEM132B |
| rs6750115 | LINC01886 | rs2931440 | ARHGEF28 | rs1421937 | ITGA2 |
| rs2200968 | LOC105376118 | rs17629792 | GALNT10 | rs2639703 | ENAH |
| rs995078 | MIR99AHG | rs651853 | LOC102723838 | rs17145082 | DNAH11 |
| rs7972608 | LETMD1 | rs10488758 | LOC105369463 | rs12342760 | LOC105376107 |
| rs7535159 | TMEM9 | rs251177 | FCHSD1 | rs310727 | SUMF1 |
| rs10864649 | ARV1 | rs4626318 | ANKRD31 | rs978927 | ADAMTS3 |
| rs17453184 | FUBP3 | rs10165154 | LRP1B | rs7975682 | IFNG-AS1 |
| rs6538025 | MGAT4C | rs9609781 | LARGE1 | rs7829124 | NRG1 |
| rs2431322 | PAM | rs1325053 | LOC401478 | rs35121625 | LINC02511 |
| rs7956547 | IGF1 | rs1532579 | CLIP1 | rs2066843 | NOD2 |
| rs11072430 | CD276 | rs1554903 | CIB4 | rs2642440 | MTARC1 |
| rs6893676 | PIK3R1 | rs2839185 | MCM3AP | rs11070592 | SEMA6D |
| rs16917735 | KIF18A | rs2665636 | LOC112268015 | rs4669 | TGFBI |
| rs41486546 | VAV3 | rs1431584 | LOC105372158 | rs16883877 | LOC107986405 |
| rs4134051 | LOC107984878 | rs10901745 | LHPP | rs17099262 | SYT16 |
| rs7655519 | NDST3 | rs434256 | CAMK4 | rs236212 | LINC00867 |
| rs1385542 | TNR | rs2183644 | LOC107985449 | rs2176235 | LOC105369838 |
| rs10131923 | MDGA2 | rs10984756 | LOC107987122 | rs13185299 | NDFIP1 |
| rs4705797 | MCC | rs676660 | PITX1-AS1 | rs12679238 | STMN2 |
| rs13402575 | LOC105373936 | rs1516699 | CCSER1 | rs2332933 | MSI2 |
| rs137205 | SEZ6L | rs4806877 | ZNF555 | rs11062885 | PARP11 |
| rs9645351 | PIFO | rs9598150 | LINC00378 | rs7022455 | TRIM14 |
| rs972449 | DELEC1 | rs954658 | TMEM108 | rs12297890 | SYT1 |
| rs2878176 | METTL24 | rs601748 | L3MBTL4 | rs9487459 | NDUFAF4 |
| rs595888 | LOC105371654 | rs626787 | USP1 | rs355909 | COBLL1 |
| rs10262410 | RPA3 | rs1641525 | ATP1B2 | rs6425398 | ASTN1 |
| rs9553604 | ATP8A2 | rs550373 | DSE | rs1371620 | LINC01934 |
| rs10888582 | RPRD2 | rs9323877 | SLC24A4 | rs8098844 | LOC105372158 |
| rs4725649 | NOBOX | rs3808422 | TRPS1 | rs11130402 | CACNA2D3 |
| rs6794676 | SRPRB | rs917158 | TMEM178B | rs6691412 | LMX1A |
| rs6471976 | LINC02842 | rs1061594 | MAP6D1 | rs934734 | SPRED2 |
| rs12796293 | METTL15 | rs11673909 | LOC102724542 | rs3749380 | GRM7 |
| rs1779301 | PKP1 | rs10772498 | ETV6 | rs10918683 | POU2F1 |
| rs8072229 | RPTOR | rs17133377 | SDK1 | rs2864755 | MTSS2 |
| rs7312364 | ADGRD1 | rs2369898 | LINC02299 | rs11661849 | FHOD3 |
| rs17624671 | ARHGAP17 | rs304262 | LOC105371843 | rs3114457 | MACC1 |
| rs12817810 | TRHDE | rs17065376 | LINC01098 | rs2514706 | RGS22 |
| rs1838059 | LINC02758 | rs16945925 | LOC105370002 | rs874083 | CAMK2A |
| rs10132818 | RGS6 | rs4545066 | C8orf37-AS1 | rs1535753 | FRMD3 |
| rs4526996 | FURIN | rs4044329 | SERGEF | rs10111296 | CASC8 |
| rs9283926 | OFCC1 | rs1484769 | STXBP4 | rs4605213 | NME2 |
| rs676236 | LOC105371654 | rs2697306 | ANKRD44 | rs11016021 | PTPRE |
| rs2075000 | CRYGN | rs11580495 | LINC01363 | rs8096797 | LOC105372092 |
| rs4917161 | ABCA13 | rs2802096 | SLC44A3-AS1 | rs16953294 | LINC02169 |
| rs7513434 | LOC107985174 | rs6546324 | LOC105374786 | rs12700960 | CHN2 |
| rs10761245 | PHF2 | rs3796362 | LIMD1 | rs2337024 | TMEM241 |
| rs9323139 | MDGA2 | rs7019136 | KLF9-DT | rs11110560 | ANO4 |
| rs13057362 | GUSBP11 | rs232086 | GTF3C1 | rs2273360 | NELFCD |
| rs3181200 | TNFSF8 | rs4924841 | ALKBH5 | rs10807008 | BTN3A1 |
| rs1554393 | LOC107984543 | rs4419846 | MTUS1 | rs11776531 | CSGALNACT1 |
| rs10819043 | GAPVD1 | rs6823546 | SORBS2 | rs1832183 | LINC00383 |
| rs13103963 | MCUB | rs11191734 | NEURL1 | rs2602141 | TP53BP1 |
| rs11938228 | TLR2 | rs4667071 | LOC107985783 | rs11761839 | HIPK2 |
| rs2038615 | SYNE1 | rs7138495 | MYBPC1 | rs990462 | FHOD3 |
| rs950777 | FHOD3 | rs2807357 | LOC105376850 | rs7398095 | ANO2 |
| rs7955732 | TRHDE | rs17382798 | PEAK1 | rs1650141 | CHST11 |
| rs2317703 | SENP7 | rs12476341 | CYBRD1 | rs174549 | FADS1 |
| rs1513826 | ACOXL | rs11016853 | MGMT | rs4730006 | LHFPL3 |
| rs11088567 | MIR99AHG | rs10186749 | HS1BP3 | rs1864207 | ERFL |
| rs1583319 | PITPNC1 | rs11105415 | LOC107984543 | rs3811862 | CDH18 |
| rs10477376 | FBXO38 | rs11009157 | ITGB1 | rs16849964 | SLC38A11 |
| rs860580 | MAP3K1 | rs4980025 | ZMIZ1 | rs2172835 | LINC02694 |
| rs3807095 | SEMA3C | rs12703774 | SLC37A3 | rs2297627 | FOXO1 |
| rs1208260 | TARID | rs6064389 | CSTF1 | rs203093 | CA10 |
| rs7955414 | ITPR2 | rs2028608 | NAV2 | rs9396888 | MIR548A1HG |
| rs2679144 | SLC10A7 | rs2155271 | LINC02698 | rs17167808 | LOC105375518 |
| rs12954678 | DSG2-AS1 | rs17169771 | NPSR1-AS1 | rs11662751 | L3MBTL4 |
| rs1024696 | RBFOX1 | rs10984743 | LOC107987122 | rs1862613 | SNX18 |
| rs11767651 | RSBN1L | rs742930 | COL10A1 | rs17746482 | ELDR |
| rs1426223 | GABRB3 | rs7776725 | FAM3C | rs2823607 | MIR99AHG |
| rs4481682 | LOC105376244 | rs946486 | ABL1 | rs2535913 | DCAF4 |
| rs10882799 | TLL2 | rs12579796 | PCED1B | rs7769927 | POLR1C |
| rs7166565 | IGF1R | rs9613287 | LOC110091768 | rs355231 | LRRC4C |
| rs1145158 | SRGAP3 | rs3804621 | DPPA4 | rs403930 | LOC101930275 |
| rs283781 | LOC101927078 | rs9355924 | PRKN | rs7499175 | PKD1L2 |
| rs269957 | NCR1 | rs12353211 | LOC105376214 | rs1467188 | NGEF |
| rs1606973 | LOC730100 | rs17047648 | LOC102723413 | rs2820001 | CDKAL1 |
| rs7841661 | SPIDR | rs230500 | NFKB1 | rs4621891 | LOC105376121 |
| rs17167482 | LRGUK | rs7766305 | LY86 | rs4318360 | LOC107985845 |
| rs8013446 | ZNF410 | rs9395005 | POLR1C | rs38488 | GARS1-DT |
| rs13374478 | VAV3 | rs4328125 | CTNNA3 | rs6900126 | LOC105377861 |
| rs9878482 | SLC6A11 | rs12726880 | LOC105378797 | rs6025714 | PMEPA1 |
| rs10799001 | CSMD2 | rs13265370 | SNTB1 | rs41498450 | LOC105377509 |
| rs7178255 | GABRB3 | rs4279007 | LOC107985484 | rs1610163 | FRMD4A |
| rs3829628 | DSG2 | rs355839 | COBLL1 | rs6460525 | AUTS2 |
| rs17799178 | KCNK13 | rs6939344 | SAMD3 | rs1904612 | CTNNA3 |
| rs513593 | CSMD2 | rs4703727 | ZBED3-AS1 | rs11743060 | SGCD |
| rs17298063 | TMEM108 | rs12253830 | LINC02646 | rs10878405 | LRRK2 |
| rs679883 | LOC105375323 | rs164283 | SLAMF1 | rs11853469 | LRRK1 |
| rs1476520 | GSDME | rs2722005 | ZBTB20 | rs3757340 | MUCL3 |
| rs17199880 | STAG1 | rs1037265 | NTM | rs17222016 | CLRN1-AS1 |
| rs155387 | CNTN6 | rs2243700 | C8orf34 | rs6541368 | SLC44A3-AS1 |
| rs17774817 | RPS29 | rs7033940 | UHRF2 | rs12136766 | RERE |
| rs12310570 | CCDC63 | rs927251 | SLC35F4 | rs11023051 | SPON1 |
| rs2505939 | BCKDHB | rs3845931 | LOC105374126 | rs1945897 | MIR4300HG |
| rs217311 | SNAP91 | rs41379551 | DGKB | rs2527031 | SEMA3A |
| rs897416 | KRT38 | rs3791035 | KDM4A | rs2060531 | ZNF385D |
| rs6444089 | NMRAL2P | rs596976 | PGM2L1 | rs7226852 | GAREM1 |
| rs10894033 | BARX2 | rs996877 | LOC105369838 | rs10869511 | OSTF1 |
| rs17598809 | COX7B2 | rs3910449 | RBFOX1 | rs17682751 | SLC4A7 |
| rs13114476 | COX7B2 | rs17319679 | TET2 | rs9360361 | ADGRB3 |
| rs10509289 | CTNNA3 | rs8036777 | ATP8B4 | rs7897426 | KCNMA1 |
| rs4653477 | CDC42BPA | rs7648309 | TKT | rs17668150 | KIAA1671 |
| rs159523 | STMN1 | rs6811695 | LOC105377401 | rs12439542 | IL16 |
| rs10898868 | ARAP1 | rs4405503 | SPRED1 | rs284267 | PEX14 |
| rs4647669 | CASP3 | rs7137586 | PTPRR | rs11264249 | KCNN3 |
| rs6783689 | SUMF1 | rs5761585 | TPST2 | rs10775471 | PIK3C3 |
| rs11978799 | FBXL13 | rs34889802 | HAPLN1 | rs17069222 | LOC105372158 |
| rs11024074 | PLEKHA7 | rs6940541 | PACRG | rs17821926 | COL17A1 |
| rs6744284 | UGT1A10 | rs880057 | LOC107986945 | rs1425889 | VWC2 |
| rs10114649 | PALM2AKAP2 | rs7804592 | LOC101927354 | rs11130791 | FHIT |
| rs2911131 | ERAP1 | rs726805 | HDAC9 | rs2994339 | SDCCAG8 |
| rs17027476 | PIFO | rs4984834 | LOC105371044 | rs10763821 | SVIL2P |
| rs242398 | PELI2 | rs742745 | LOC105372620 | rs7736549 | THBS4 |
| rs2901277 | DPP10 | rs17368044 | PKHD1L1 | rs17523814 | CNTNAP2 |
| rs7305694 | PIK3C2G | rs175490 | ZC2HC1C | rs12624154 | LRRFIP1 |
| rs17746145 | LOC105377262 | rs10519250 | ATP8B4 | rs6561112 | ENOX1 |
| rs878744 | LOC102723803 | rs7830377 | LINC00535 | rs12313261 | KCNC2 |
| rs7832096 | TNKS | rs2518390 | LINC02762 | rs3112639 | LOC105371070 |
| rs4756026 | SLC35C1 | rs2062331 | MSRA | rs405667 | LINC02703 |
| rs6481738 | LOC105376481 | rs6723510 | LOC730100 | rs1345591 | SLIT3 |
| rs7736793 | COMMD10 | rs7634185 | SCHIP1 | rs2053568 | SEC14L1 |
| rs7274531 | SLC24A3 | rs2935542 | C1QA | rs4848919 | CNTNAP5 |
| rs17468203 | LOC107984005 | rs7190850 | RBFOX1 | rs3786385 | PTPRM |
| rs17818670 | DENND4C | rs4132664 | SLC35F1 | rs912790 | LINC00572 |
| rs6498142 | CLEC16A | rs11777119 | SARAF | rs4959787 | PSMG4 |
| rs2265458 | LINC02334 | rs12445562 | LOC105371393 | rs10861570 | SYT1 |
| rs4920580 | PADI3 | rs730646 | WHRN | rs10502161 | NCAM1 |
| rs2664122 | SRGAP3 | rs515291 | SLC2A13 | rs2740485 | ABCA1 |
| rs10041072 | IRGM | rs12521915 | ITGA2 | rs4799140 | PARD6G |
| rs10118714 | LOC105375974 | rs303171 | IFIT5 | rs411648 | MACIR |
| rs2432175 | LOC105379089 | rs499459 | WTAPP1 | rs331096 | FBN2 |
| rs1006752 | DTNB | rs4748387 | HACD1 | rs6883434 | PRELID2 |
| rs152134 | PPIP5K2 | rs2139539 | COL16A1 | rs7921150 | LOC105376360 |
| rs2076756 | NOD2 | rs1167244 | ERC2 | rs3741028 | IMMP1L |
| rs4109733 | MICU3 | rs2051058 | ZNF227 | rs1887708 | GPC6 |
| rs17167492 | LRGUK | rs1031125 | LOC105377331 | rs17406994 | NCALD |
| rs427608 | ZNF230 | rs2215617 | NEUROD6 | rs2857435 | TRIM40 |
| rs10473086 | LINC02107 | rs10490820 | SRGAP3 | rs12584825 | CLYBL |
| rs9950563 | FHOD3 | rs7959277 | TMEM132B | rs883724 | DAGLA |
| rs12352699 | LOC105376107 | rs1006334 | POT1-AS1 | rs7964411 | CCDC38 |
| rs12357998 | CTNNA3 | rs12110370 | SLC35F1 | rs4496905 | LOC107986841 |
| rs1564796 | AATF | rs1529497 | SCAMP1 | rs7038903 | SVEP1 |
| rs3858253 | LOC107984219 | rs2585192 | MEGF10 | rs4722988 | NOD1 |
| rs4833376 | LOC102724210 | rs17729477 | ITPR1 | rs4579186 | IGFBP7-AS1 |
| rs6787687 | LSAMP | rs2200218 | TRPS1 | rs17512199 | NRXN1 |

**S3 Table. Risk genes of group P001.**

| **SNP** | **Gene** | **SNP** | **Gene** | **SNP** | **Gene** |
| --- | --- | --- | --- | --- | --- |
| rs2108978 | AKAP10 | rs2431322 | PAM | rs2744374 | DSP |
| rs2381971 | PTPRD | rs3943074 | SUMF1 | rs2287144 | TOX3 |
| rs4519393 | MC2R | rs2439614 | KLHL1 | rs10769118 | TRIM5 |
| rs17109102 | NRXN3 | rs302946 | UBE2K | rs7016691 | NRG1 |
| rs10185950 | RND3 | rs6589847 | GRIK4 | rs6795481 | SUMF1 |
| rs9945680 | ZBTB14 | rs521069 | MPPED2 | rs41496145 | LRGUK |
| rs12325711 | SHISA6 | rs7781054 | RPA3 | rs238328 | DGKH |
| rs25112 | SYN3 | rs2935651 | CTBP2 | rs17383719 | PBX1 |
| rs10503913 | NRG1 | rs16932962 | TTC39B | rs971215 | SNAP91 |
| rs1946127 | RBFOX1 | rs10846883 | TMEM132B | rs2741919 | MEFV |
| rs11104913 | KITLG | rs4797825 | MC2R | rs11971451 | LRGUK |
| rs757747 | SEMA3A | rs11237796 | TENM4 | rs743904 | TNR |
| rs12213597 | FAM184A | rs2323587 | BARX2 | rs10490051 | SLC8A1 |
| rs361021 | IQSEC1 | rs10780196 | CACNA1B | rs4438608 | FGF12 |
| rs4149269 | ABCA1 | rs16968438 | ASIC2 | rs11101442 | WDFY4 |
| rs1555890 | TGFBR3 | rs5754259 | SYN3 | rs10821716 | ANK3 |
| rs3956806 | KSR2 | rs9557205 | UBAC2 | rs333950 | CSF1 |
| rs17128052 | GCH1 | rs17016168 | SNCA | rs6056855 | PAK5 |
| rs7153625 | NRXN3 | rs1683348 | FHIT | rs700469 | PKP1 |
| rs1863967 | ADAMTS16 | rs659991 | DGKH |  |  |
| rs17188506 | KCNH1 | rs1748041 | PADI4 |  |  |

**S4 Table. Risk genes of group P005.**

| **SNP** | **Gene** | **SNP** | **Gene** | **SNP** | **Gene** |
| --- | --- | --- | --- | --- | --- |
| rs10771012 | SOX5 | rs745696 | ZNF219 | rs7780487 | DPP6 |
| rs984066 | DPP10 | rs2092867 | NFIA | rs11090417 | MYO18B |
| rs2240920 | ITIH3 | rs2197714 | EPHB1 | rs13438514 | SKAP2 |
| rs4765905 | CACNA1C | rs4263970 | EPHB2 | rs16897440 | VPS13B |
| rs2042794 | SYN3 | rs1861474 | MSI2 | rs655970 | KMO |
| rs1016351 | CWF19L2 | rs11720582 | ADAMTS9-AS2 | rs760233 | TTLL5 |
| rs7899110 | RBM17 | rs7544440 | GREM2 | rs8176044 | KEL |
| rs5747087 | CECR2 | rs5765490 | FBLN1 | rs12233096 | HDAC4 |
| rs1469853 | FGF14 | rs2834238 | DONSON | rs4656538 | POU2F1 |
| rs12706898 | NRF1 | rs5747145 | CECR2 | rs208021 | PCM1 |
| rs2594135 | FHIT | rs17167543 | LRGUK | rs457587 | C5orf67 |
| rs193858 | CDHR3 | rs6593171 | GRB10 | rs4978934 | SVEP1 |
| rs930758 | UCHL1 | rs7984662 | GPC5 | rs4484586 | DNAH11 |
| rs17503541 | MSH2 | rs9557193 | GPR183 | rs17753220 | TPM1 |
| rs2292641 | PGS1 | rs9303394 | MSI2 | rs1059502 | CPB1 |
| rs2867880 | CLEC16A | rs12910386 | ACSBG1 | rs6977749 | PTN |
| rs10779279 | ESRRG | rs324139 | POLR1C | rs2281719 | GALNT2 |
| rs7146075 | NRXN3 | rs7971179 | FAR2 | rs76674 | BCL11A |
| rs4765902 | CACNA1C | rs12444562 | JPH3 | rs134661 | KREMEN1 |
| rs466639 | RXRG | rs4672393 | BCL11A | rs7777879 | RPA3 |
| rs9472138 | POLR1C | rs2471857 | DRD2 | rs1804690 | HYOU1 |
| rs4894769 | PLD1 | rs12582879 | GRIP1 | rs4936749 | UBASH3B |
| rs514636 | LAMP3 | rs6446159 | FHIT | rs4308217 | CD86 |
| rs2238056 | CACNA1C | rs2836532 | ERG | rs1227053 | CDH23 |
| rs1565073 | DOCK10 | rs494958 | SRD5A1 | rs2834213 | IFNGR2 |
| rs615837 | NDST3 | rs9850375 | ADCY5 | rs1549759 | CDK5 |
| rs1179494 | FEZ2 | rs4112527 | ITGBL1 | rs9679095 | COL4A3 |
| rs17167639 | LRGUK | rs615967 | RAD18 | rs11935252 | TLR2 |
| rs8050643 | CDH13 | rs12874185 | GPC5 | rs11077154 | RBFOX1 |
| rs2466296 | SLC30A8 | rs6425658 | STX6 | rs4413512 | ARHGEF28 |
| rs12157657 | GUSBP11 | rs4789291 | UBE2O | rs3814821 | IFI27 |
| rs910050 | TSBP1 | rs403029 | GATA3 | rs2884208 | HPCAL1 |
| rs9850123 | FGF12 | rs17315775 | SKAP2 | rs212857 | SKAP2 |
| rs4328757 | TRANK1 | rs2154487 | GRIK1 | rs5761272 | MYO18B |
| rs9482771 | RSPO3 | rs2243834 | RAB10 | rs7972260 | TMEM132B |
| rs4293434 | MYO1D | rs2488556 | VAV2 | rs1421937 | ITGA2 |
| rs702465 | BOLA3 | rs2122576 | SMC2 | rs2639703 | ENAH |
| rs2945401 | KSR1 | rs7117858 | LINC02751 | rs17145082 | DNAH11 |
| rs17453184 | FUBP3 | rs8088134 | DLGAP1 | rs310727 | SUMF1 |
| rs2431322 | PAM | rs2931440 | ARHGEF28 | rs7829124 | NRG1 |
| rs7956547 | IGF1 | rs10165154 | LRP1B | rs2066843 | NOD2 |
| rs11072430 | CD276 | rs1532579 | CLIP1 | rs2642440 | MTARC1 |
| rs6893676 | PIK3R1 | rs434256 | CAMK4 | rs11070592 | SEMA6D |
| rs41486546 | VAV3 | rs3808422 | TRPS1 | rs17099262 | SYT16 |
| rs7655519 | NDST3 | rs10772498 | ETV6 | rs13185299 | NDFIP1 |
| rs1385542 | TNR | rs2697306 | ANKRD44 | rs12679238 | STMN2 |
| rs137205 | SEZ6L | rs232086 | GTF3C1 | rs2332933 | MSI2 |
| rs10262410 | RPA3 | rs7138495 | MYBPC1 | rs7022455 | TRIM14 |
| rs1779301 | PKP1 | rs12476341 | CYBRD1 | rs12297890 | SYT1 |
| rs8072229 | RPTOR | rs11016853 | MGMT | rs9487459 | NDUFAF4 |
| rs4526996 | FURIN | rs10186749 | HS1BP3 | rs355909 | COBLL1 |
| rs9283926 | OFCC1 | rs11009157 | ITGB1 | rs934734 | SPRED2 |
| rs10761245 | PHF2 | rs946486 | ABL1 | rs10918683 | POU2F1 |
| rs13057362 | GUSBP11 | rs9355924 | PRKN | rs874083 | CAMK2A |
| rs10819043 | GAPVD1 | rs230500 | NFKB1 | rs4605213 | NME2 |
| rs11938228 | TLR2 | rs9395005 | POLR1C | rs12700960 | CHN2 |
| rs2038615 | SYNE1 | rs355839 | COBLL1 | rs2273360 | NELFCD |
| rs1513826 | ACOXL | rs164283 | SLAMF1 | rs10807008 | BTN3A1 |
| rs10477376 | FBXO38 | rs927251 | SLC35F4 | rs2602141 | TP53BP1 |
| rs860580 | MAP3K1 | rs41379551 | DGKB | rs174549 | FADS1 |
| rs2679144 | SLC10A7 | rs3791035 | KDM4A | rs2172835 | LINC02694 |
| rs1024696 | RBFOX1 | rs3910449 | RBFOX1 | rs2297627 | FOXO1 |
| rs11767651 | RSBN1L | rs17319679 | TET2 | rs1862613 | SNX18 |
| rs1426223 | GABRB3 | rs7648309 | TKT | rs7769927 | POLR1C |
| rs7166565 | IGF1R | rs4405503 | SPRED1 | rs355231 | LRRC4C |
| rs269957 | NCR1 | rs726805 | HDAC9 | rs7499175 | PKD1L2 |
| rs17167482 | LRGUK | rs2062331 | MSRA | rs1467188 | NGEF |
| rs13374478 | VAV3 | rs2935542 | C1QA | rs2820001 | CDKAL1 |
| rs7178255 | GABRB3 | rs7190850 | RBFOX1 | rs10878405 | LRRK2 |
| rs17774817 | RPS29 | rs4132664 | SLC35F1 | rs12136766 | RERE |
| rs2505939 | BCKDHB | rs730646 | WHRN | rs2527031 | SEMA3A |
| rs217311 | SNAP91 | rs515291 | SLC2A13 | rs7897426 | KCNMA1 |
| rs10894033 | BARX2 | rs12521915 | ITGA2 | rs12439542 | IL16 |
| rs4653477 | CDC42BPA | rs499459 | WTAPP1 | rs11264249 | KCNN3 |
| rs159523 | STMN1 | rs7959277 | TMEM132B | rs10775471 | PIK3C3 |
| rs4647669 | CASP3 | rs12110370 | SLC35F1 | rs17821926 | COL17A1 |
| rs6783689 | SUMF1 | rs1529497 | SCAMP1 | rs11130791 | FHIT |
| rs6744284 | UGT1A10 | rs2585192 | MEGF10 | rs7736549 | THBS4 |
| rs10114649 | PALM2AKAP2 | rs2200218 | TRPS1 | rs17523814 | CNTNAP2 |
| rs2911131 | ERAP1 | rs11214512 | NCAM1 | rs12624154 | LRRFIP1 |
| rs2901277 | DPP10 | rs2482814 | PCDH15 | rs6561112 | ENOX1 |
| rs7305694 | PIK3C2G | rs5024299 | PIEZO2 | rs12313261 | KCNC2 |
| rs7832096 | TNKS | rs3790999 | COL6A3 | rs2053568 | SEC14L1 |
| rs7736793 | COMMD10 | rs7181486 | IREB2 | rs4848919 | CNTNAP5 |
| rs7274531 | SLC24A3 | rs329674 | IGSF9B | rs10861570 | SYT1 |
| rs6498142 | CLEC16A | rs16966655 | SPRED1 | rs10502161 | NCAM1 |
| rs4920580 | PADI3 | rs6728886 | ITGA4 | rs2740485 | ABCA1 |
| rs10041072 | IRGM | rs17100876 | RHOJ | rs411648 | MACIR |
| rs2076756 | NOD2 | rs11021901 | GALNT18 | rs17406994 | NCALD |
| rs17167492 | LRGUK | rs6750599 | BCL2L11 | rs7038903 | SVEP1 |
| rs1564796 | AATF | rs16984549 | GRIK1 | rs4722988 | NOD1 |
| rs6787687 | LSAMP | rs10506625 | TSPAN8 |  |  |
